# Taming interfacial electronic properties of platinum nanoparticles on vacancy-abundant boron nitride nanosheets for enhanced catalysis

**DOI:** 10.1038/ncomms15291

**Published:** 2017-06-09

**Authors:** Wenshuai Zhu, Zili Wu, Guo Shiou Foo, Xiang Gao, Mingxia Zhou, Bin Liu, Gabriel M. Veith, Peiwen Wu, Katie L. Browning, Ho Nyung Lee, Huaming Li, Sheng Dai, Huiyuan Zhu

**Affiliations:** 1 School of Chemistry and Chemical Engineering, Institute for Energy Research, Jiangsu University, Zhenjiang 212013, China; 2 Chemical Sciences Division, Oak Ridge National Laboratory, Oak Ridge, Tennessee 37831, USA; 3 Materials Science and Technology Division, Oak Ridge National Laboratory, Oak Ridge, Tennessee 37831, USA; 4 Department of Chemical Engineering, Kansas State University, Manhattan, Kansas 66506, USA; 5 Institute for Energy Research, Jiangsu University, Zhenjiang 212013, China

## Abstract

Taming interfacial electronic effects on Pt nanoparticles modulated by their concomitants has emerged as an intriguing approach to optimize Pt catalytic performance. Here, we report Pt nanoparticles assembled on vacancy-abundant hexagonal boron nitride nanosheets and their use as a model catalyst to embrace an interfacial electronic effect on Pt induced by the nanosheets with N-vacancies and B-vacancies for superior CO oxidation catalysis. Experimental results indicate that strong interaction exists between Pt and the vacancies. Bader charge analysis shows that with Pt on B-vacancies, the nanosheets serve as a Lewis acid to accept electrons from Pt, and on the contrary, when Pt sits on N-vacancies, the nanosheets act as a Lewis base for donating electrons to Pt. The overall-electronic effect demonstrates an electron-rich feature of Pt after assembling on hexagonal boron nitride nanosheets. Such an interfacial electronic effect makes Pt favour the adsorption of O_2_, alleviating CO poisoning and promoting the catalysis.

## Abstract (web summary)

Tuning electronic properties of metallic catalysts is a useful way to improve their activity, however control over metal-support interactions is still challenging. Here the authors report a vacancy-induced interfacial electronic effect for Pt assembled on vacancy-abundant *h*-BN nanosheets leading to superior CO oxidation catalysis.

Tuning interfacial electronic properties has emerged as a vital topic in heterogeneous catalysis because of its paramount importance to both practical usage and fundamental understanding of nanoparticle (NP) catalysts^1^,^2^. Several strategies have been applied to tune the electronic properties of metal nanocatalysts: (1) constructing a multimetallic, that is, binary^3^,^4^,^5^, ternary^6^,^7^, core/shell^8^,^9^,^10^,^11^ or dumbbell structure^12^, where the electronic structure of a catalytically active metal can be modified by other components; (2) using organic ligand modifiers with a desirable electron-donating/withdrawing property on a metal catalyst^1^,^13^,^14^,^15^,^16^; (3) creating metal-support interactions^17^,^18^,^19^,^20^,^21^, including the strong metal support interaction as well as employing redox-active supports and thereby introducing electronic/geometric modifications on metal catalysts^22^,^23^. Although the first two strategies have been well-documented in the literature, renewed interest on the metal-support interface has only been witnessed in recent years.

Supported Pt NP catalysts have continuously drawn broad and increasing attention because of their unique catalytic activity for a large number of important chemical reactions, for example, CO oxidation^24^,^25^, small molecule hydrogenation^1^, oxygen reduction^26^,^27^ and hydrogen evolution^28^. However, a challenge remains regarding how to arbitrarily tune the electronic structure of Pt for enhanced catalysis. Taking CO oxidation as an example—on a Pt surface, owing to the strong binding of CO, the O_2_ activation is usually blocked; therefore a redox-active support (TiO_2_ (ref. 29), CeO_2_ (refs 30, 31), FeO_x_ (ref. 32)) that can transfer its lattice oxygen to the Pt catalyst's surface is a prerequisite for efficient CO oxidation. In the case of a non-redox active support (SiO_2_, Al_2_O_3_ and C), the Pt activity for CO oxidation is usually very low with a full conversion temperature (*T*_100_) above 150 °C (ref. 32) (Supplementary Table 1). Here our Pt NPs on a non-redox active hexagonal boron nitride (*h*-BNNs) support with low loading (Pt content=1.18 wt.% by inductively coupled plasma optical emission spectroscopy determination) can catalyse CO oxidation with a *T*_100_ below 70 °C. Meanwhile, there is to our knowledge no report of Pt NPs assembled on vacancy-abundant hexagonal boron nitride nanosheets (*h*-BNNS) to embrace an interfacial electronic effect on Pt induced by 2-dimensional (2D) *h*-BNNS with N-vacancies (N_V_) and B-vacancies (B_V_) for superior CO oxidation catalysis. Recently, we have reported a gas exfoliation method to prepare ultrathin *h*-BNNS with high surface area and abundant edges and B/N vacancies^33^, which could serve as an ideal non-redox active support for Pt NPs. These exfoliated *h*-BNNS are characterised by fewer layers, smaller lateral size and more B/N defects including a high exposure of B_V_ and N_V_ edges than bulk *h*-BN (Fig. 1). Moreover, compared with covalent C–C bonding in graphene, the B–N bonding in *h*-BNNS displays partially ionic character, making *h*-BNNS a prominent electronic-tuning support to interact with Pt^23^.

In this work, we demonstrate an interfacial electronic effect on Pt NP catalysts modulated by the vacancy-abundant *h*-BNNS for superior CO oxidation performance. The Pt NPs supported on *h*-BNNS (denoted as Pt/*h*-BNNS) demonstrate a high CO oxidation activity. The durability test shows that the Pt/*h*-BNNS catalyst maintains 100% CO conversion efficiency after 50 h at 75 °C. The interfacial electronic effect is found to stem primarily from the interaction between Pt and B_V_ as well as Pt and N_V_. Both electron energy loss spectroscopy (EELS) and density functional theory (DFT) calculations suggest that there exists a strong interaction between the Pt and B/N vacancies. EELS results indicate that the interaction between Pt and the N_V_ is predominant. According to Bader charge analysis, we find that with Pt on B_V_, the *h*-BNNS serves as a Lewis acid to accept electrons from Pt, whereas when Pt interacts with N_V_, the *h*-BNNS acts as a Lewis base for donating electrons to Pt. The overall-electronic effect evaluated by *in situ* diffuse reflectance infrared spectroscopy (DRIFTS) demonstrates that Pt is electronic rich with Pt/Nv as the primary interaction. Consequently, such an interfacial electronic effect makes Pt NPs favour the adsorption of O_2_, alleviating CO poisoning and promoting the catalysis. Our discovery gives understanding of the interfacial electronic effects between Pt and a non-redox active support and may provide an important strategy to tailor the Pt electronic structure for enhanced catalysis.

## Results

### Synthesis and characterization of Pt/*h*-BNNS

**Figure 2:**
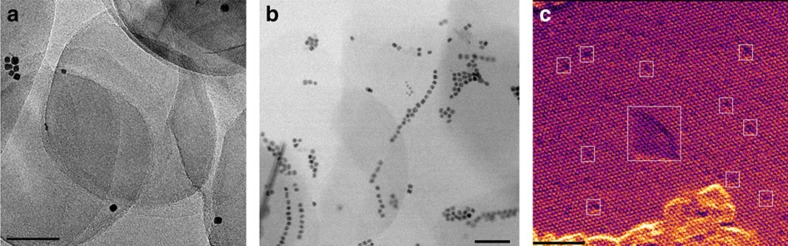
Representative TEM and STEM images. (**a**) TEM of Pt/*h*-BNNS. Scale bar, 50 nm. (**b**) STEM of Pt/*h*-BNNS. Scale bar, 50 nm. (**c**) High-resolution STEM of *h*-BNNS with vacancies, as indicated by the boxes. Scale bar, 2 nm.

The *h*-BNNS and Pt NPs were synthesized according to previous reports^33^,^34^. The preparation of Pt/*h*-BNNS catalyst follows a sonication-assisted colloidal deposition and the process is presented in the Experimental section. Figure 2a and Supplementary Fig. 1 show the representative bright-field transmission electron microscopy (TEM) and scanning transmission electron microscopy (STEM) images of exfoliated few-layer *h*-BNNS with high exposure edge and 5±0.5 nm cubic Pt NPs. X-ray diffraction pattern indicates that the Pt NPs have the face-centred cubic structure (Supplementary Fig. 2), and the size estimated from Scherrer equation is consistent with the results from TEM and STEM observations. The (111) peak is much stronger than the (200) peak, demonstrating a three-dimensional randomly oriented assembly.

**Figure 3:**
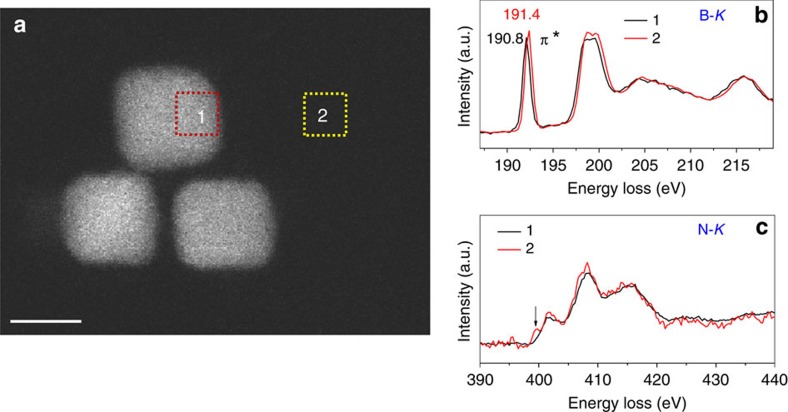
EELS analysis of Pt/*h*-BNNS. (**a**) Annular dark-field (ADF) STEM image of Pt/*h*-BNNS indicating the mapping region: 1. The centre of Pt/*h*-BNNS overlapping region; 2. h-BNNS matrix. (**b**) B-*K* EELS profile and (**c**) N-*K* EELS profile mapped from regions 1 and 2.

Bright-field TEM (Fig. 2a) and STEM (Fig. 2b) images of the Pt/*h*-BNNS demonstrate that the cubic Pt NPs embedded at *h*-BNNS sitting mostly on the edge vacancies of the support. As seen by TEM and STEM, *h*-BNNS present an ultra-thin-layered structure with abundant edges formed during exfoliation. This ultrathin feature is also proved by atomic force microscopy (AFM) (Supplementary Fig. 3) and the height of *h*-BNNS is 2–3 nm. More importantly, abundant defects consisting of either N_V_ or B_V_ were observed by high-resolution STEM (Fig. 2c). To investigate the interaction between Pt NPs and *h*-BNNS, we performed EELS to map the individual B and N in the Pt/*h*-BNNS sample, observing a chemical shift of B-K edge in its pre-peak depending only on the distance to the Pt particles (Supplementary Fig. 4 and Supplementary Movie 1). The B-K edge profiles acquired from the *h*-BNNS matrix region (2) and from the centre of the Pt/*h*-BNNS overlapping region (1) are shown in Fig. 3a. Compared with the B-K edge obtained from the (2) site, we find that the pre-peak (π*) of B-K downshifts at the (1) site, indicating that there are B-terminated N_V_ defects interacting with the Pt NPs (Fig. 3b)^35^. Supplementary Movie 1 further proves that this downshift of the pre-peak of B-K is ubiquitous depending only on the distance to Pt NPs. This is consistent with the formation of an additional peak at the threshold of N-K edge at (2) site^36^, as shown by the arrow in Fig. 3c. The additional peak appears relatively weak, although after careful examination we did not observe it in the pure BN matrix region. These results indicate that Pt NPs may prefer to be embedded on B-terminated N_V_ of *h*-BNNS instead of staying on N-terminated B_V_ of *h*-BNNS.

### Catalytic activity for CO oxidation

**Figure 4:**
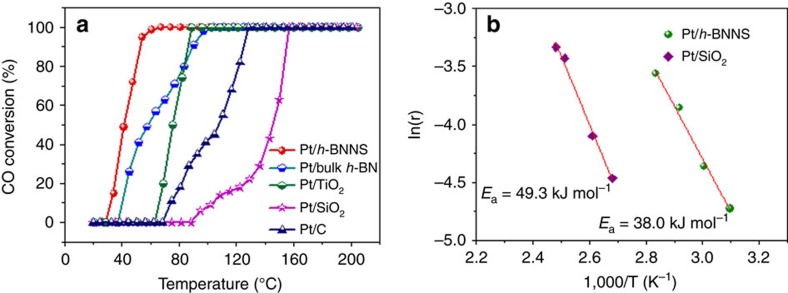
CO oxidation activity of Pt/*h*-BNNS. (**a**) CO oxidation light-off curves for the Pt/*h*-BNNS, Pt/bulk *h*-BN, Pt/TiO_2_, Pt/SiO_2_ and Pt/C, m(catalyst)=30 mg, CO flow rate 10 ml min^−1^. (**b**) The apparent activation energies (*E*_a_) of Pt/*h*-BNNS and Pt/SiO_2_, m(catalyst)=5 mg, CO flow rate 10 ml min^−1^.

The catalytic oxidation of CO as a probe reaction was evaluated over as-prepared catalysts in a temperature-programmed mode. The catalysts were packed into a U-shaped quartz tube (inner diameter=4 mm), sealed by quartz wool and treated at 200 °C for 1 h to activate the surface. CO oxidation was then performed with a gas stream consisting of 1% CO (balance air, <4 p.p.m. water) at a space velocity (SV) of 1667 Lg^−1^_Pt_ h^−1^. As shown in Fig. 4a, the Pt/*h*-BNNS (1.18 wt.% Pt content, *h*-BNNS 278 m^2^ g^−1^) displays the highest CO oxidation activity and readily converts 100% CO starting from 67 °C. As a comparison, Pt NPs on various non-redox active supports with the similar Pt content (1.15–1.21 wt.%), including bulk *h-*BN (10 m^2^ g^−1^), silica fumed (SiO_2_, 280 m^2^ g^−1^) and acetylene black (C, 260 m^2^ g^−1^) are also investigated and their corresponding *T*_100_ is 104, 156 and 128 °C at a similar SV, respectively. Even with the redox-active support TiO_2_ (P_25_, 65 m^2^ g^−1^), the *T*_100_ was found to be 88 °C at a similar SV. This catalytic performance indicates that *h-*BNNS can serve as an excellent support for Pt NPs. This CO oxidation activity is also much higher than the reported redox-active support TiO_2_-supported Pt nanocrystals with a *T*_100_ of 100 °C and non-redox active support Pt/SiO_2_ with a *T*_100_ of 150 °C^24^,^37^,^38^. The Pt/*h*-BNNS exhibits negligible CO oxidation activity only at temperatures below 35 °C, which is much lower than that of the active support TiO_2_ (69 °C) and the inert supports SiO_2_ (95 °C) and C (75 °C) under the same reaction conditions. The apparent activation energies (*E*_a_) are derived with a SV of 10^4^ L g^−1^_Pt_ h^−1^ on Pt/*h*-BNNS and Pt/SiO_2_. Pt/*h*-BNNS has an *E*_a_ value of 38.0 kJ mol^−1^, lower than that of the inert support SiO_2_ (49.3 kJ mol^−1^) (Fig. 4b). For long-time stability study, a time-on-stream test was performed on the Pt/*h*-BNNS, and the results in Supplementary Fig. 5 indicated that the catalyst maintained 100% CO conversion efficiency after 50 h at 75 °C. 75, 50 and 25% CO conversions over the Pt/*h*-BNNS were also achieved at 49, 45 and 39 °C after 15, 20 and 15 h test.

### Interfacial electronic properties of Pt/*h*-BNNS

**Figure 5:**
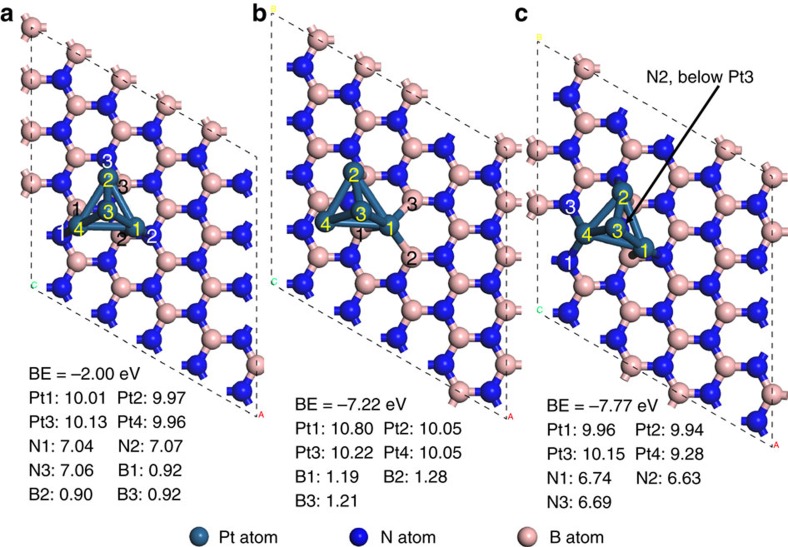
Optimized structures and valence electrons of pyramidal Pt_4_ cluster on *h-*BNNS. (**a**) Pt_4_ cluster on clean, vacancy-free *h*-BNNS. (**b**) Pt_4_ cluster *h*-BNNS with Nv. (**c**) Pt_4_ cluster on *h*-BNNS with Bv.

To prove the Pt/*h*-BNNS interface impact on the catalytic activity, we performed measurements of Pt NPs and *h*-BNNS alone under the same reaction conditions (Supplementary Fig. 6) and found *h*-BNNS has no catalytic activity for CO oxidation under 200 °C. The Pt NPs exhibit negligible CO oxidation activity at temperatures below 150 °C and converts 100% CO starting from 229 °C, which is much higher than that of Pt/*h*-BNNS. These results indicate that the enhancement effect is from the interfacial coupling between Pt and *h*-BNNS. To further understand how the Pt/*h*-BNNS interface impacts on the catalytic activity, periodic DFT calculations, employing the Generalized Gradient Approximation-PBE functional, were performed to optimize the Pt_4_ particle structures, and analyse the charge transfers at the interface of Pt/*h*-BNNS using Bader charge analysis on pristine *h*-BNNS, and *h*-BNNS with Nv and Bv single-point defects. The optimized geometries and calculated valence electrons on Pt and nearby B and N atoms are shown in Fig. 5. Other geometries (for example, flat Pt_4_) were also considered and are presented in Supplementary Figs 7 and 8. The 4-atom Pt_4_ cluster prefers a 3D tetrahedral geometry on *h*-BNNS. On the pristine h-BNNS, the charge transfer between Pt and *h*-BNNS is negligible as shown in Fig. 5a. The cluster binding energy (BE) is −2.00 eV. In the presence of Nv and Bv, the cluster preferentially binds at the defective sites, with respective BEs of −7.22 eV and −7.77 eV, by forming corresponding Pt–B and Pt–N bonds. The increase of BEs clearly indicates that vacancy sites have the role of enhancing the adsorptions and stabilising Pt particles for CO oxidation. Bader charge analyses confirmed that there exists an interfacial charge transfer between the adsorbed cluster and *h*-BNNS. At the N_V_ site (B termination) (Fig. 5b), charge transfers from *h*-BNNS to Pt, resulting in a net gain of 0.80 e on the Pt-1 atom; whereas at the B_V_ site (N termination, Fig. 5c), charge transfers away from Pt, resulting in a net loss of 0.72 e on the Pt-4 atom. A larger Pt_10_ cluster, with an optimized tetrahedral geometry was also considered, and Bader charge analyses showed that the Pt atoms at the defect sites would also gain and lose net charges at the Nv and Bv point defect sites, respectively (Supplementary Fig. 9).

**Figure 6:**
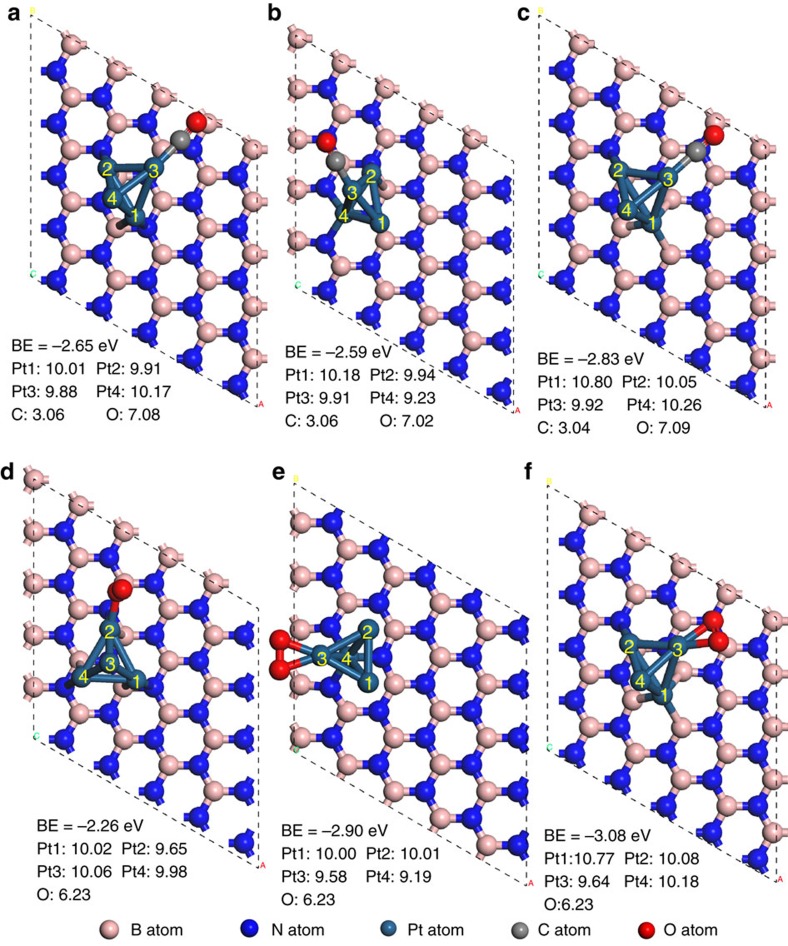
Most stable configuration of CO and O_2_ adsorption and binding energies on Pt_4_ cluster. CO adsorption: (**a**) Pt_4_ cluster on clean, vacancy-free *h*-BNNS. (**b**) Pt_4_ cluster *h*-BNNS with Bv. (**c**) Pt_4_ cluster on *h*-BNNS with Nv. O_2_ adsorption: (**d**) Pt_4_ cluster on clean, vacancy-free *h*-BNNS. (**e**) Pt_4_ cluster *h*-BNNS with Bv. (**f**) Pt_4_ cluster on *h*-BNNS with Nv.

On the Pt_4_ cluster supported on the defect-free *h*-BNNS, CO binds at the top site (Fig. 6a), with a BE_(CO)_ of −2.65 eV. On the cluster adsorbed at the Bv defective site (charge transferred away from Pt), CO binds in its most stable configuration at the Pt-3 atom, with a BE of −2.59 eV (Fig. 6b), weaker than that on the pristine Pt/*h*-BNNS. In contrast, CO binds more strongly on the cluster supported on the *h*-BNNS with Nv, with a BE of −2.83 eV than on the pristine *h*-BNNS (Fig. 6c). Moreover, the BEs of O_2_ on the Pt_4_ cluster are stronger with both Bv (−2.90 eV) (Fig. 6e) and Nv (−3.08 eV) (Fig. 6f) than that on the defect-free Pt/*h*-BNNS (−2.26 eV) (Fig. 6d). The optimized geometries and BEs of CO and O_2_ on Pt_10_ tetrahedral cluster are also considered on pristine *h*-BNNS, as well as *h*-BNNS with Nv and Bv point defects (Supplementary Fig. 10). The structural deformation on this Pt_10_ cluster is noticeably to a reduced extent. CO adsorbs on the top of the Pt atom in the edge of the cluster supported on the pristine *h*-BNNS and that with the Nv point defect. With the Bv defect, CO binds on the bridge site closer to the cluster and *h*-BNNS interface. It is also noted that the BE_(CO)_ increases near the Nv defect, accompanied by net charge gain on the cluster, whereas the BE_(CO)_ decreases near the Bv defect, accompanied by net charge loss on the cluster. O_2_ prefers to adsorb at the bridge site along the cluster edge. The BEs of O_2_ adsorptions are stronger regardless of the types of vacancy. The trends for both CO and O_2_ adsorption are consistent with the findings on smaller clusters. Hence, first-principles calculations support the fact that the electronic effects on Pt induced by vacancy-abundant *h*-BNNS could faciliate O_2_ adsorption and activation, thus enhance CO oxidation, which is in accordance with the findings by DFT calculations in the previous reports^39^,^40^. This observation also explains why bulk *h*-BN (with smaller surface area 10 m^2^g^−1^, less edges and vacancies) performs inferior to *h*-BNNS (278 m^2^g^−1^, abundant of edges and vacancies)^33^ (Supplementary Fig. 11).

## Discussion

**Figure 7:**
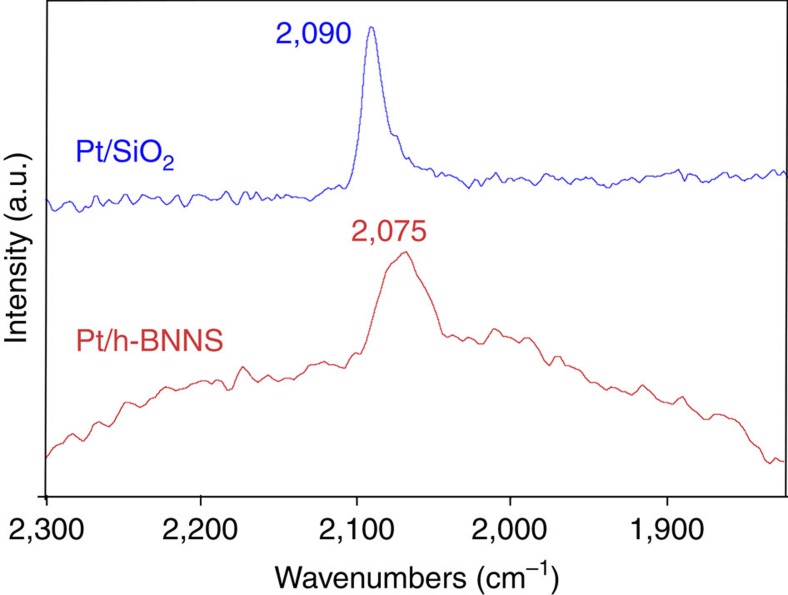
*In situ* FTIR spectra of CO adsorbed on Pt/*h-*BNNS and Pt/SiO_2_ at room temperature. Temperature=25 °C. Features from gas phase CO have been subtracted.

As mentioned above, the charge transfer was from *h*-BNNS to Pt on Nv and the corresponding opposite direction on Bv. Since both Nv and Bv are present in *h*-BNNS, to characterize the overall charge transfer between Pt and *h*-BNNS, *in situ* DRIFTS of CO adsorption was employed to probe the surface electronic state of Pt/*h*-BNNS and Pt/SiO_2_ (non-redox active support). The *in situ* DRIFTS spectra in Fig. 7 show that the linearly adsorbed CO on Pt/*h*-BNNS gives an absorption at 2,075 cm^−1^, ∼15 cm^−1^ lower than that on Pt/SiO_2_. This redshift of the CO frequency indicates that the surfaces of Pt NPs are more negatively charged on *h*-BNNS than on SiO_2_. It suggests that the overall electron transfer is from *h*-BNNS to Pt NPs^41^, consistent with our EELS result that Pt preferably interacts with B atoms at N_V_, where B atoms at N_V_ tend to transfer charge to Pt (0.8 eV) based on Bader charge analysis (Fig. 5b). X-ray photoelectron spectroscopy (XPS) was performed to probe the change in the BE of Pt to further investigate the charge transfer between Pt and *h*-BNNS. XPS data were collected for the Pt/*h*-BNNS catalyst in Supplementary Fig. 12. The data showed typical *h*-BNNS spectra with little to no surface oxidation evident in the N1s and B1s plots. This also indicates that the catalytic activity enhancement is not due to the creation of B-O functionality at the support-Pt interface. The Pt 4f_7/2_ data show the presence of two unique Pt species with BEs of 70.73 and 72.56 eV corresponding to Pt^o^ and Pt^2+^, respectively, with a relative ratio of 4:1. The Pt 4f_5/2_ data are shifted by 3.3 eV relative to the Pt 4f_7/2_ value consistent with the presence of two Pt^o^ and Pt^2+^ species (relative ratio of 3.9:1). This suggests that the majority of the Pt is in the reduced state consistent with what is required for CO oxidation. XPS data collected for the non-redox active support of Pt/SiO_2_ sample also indicated a signal for the Pt species consistent with Pt^0^ and Pt^2+^ species. However, the corresponding relative ratio of Pt^0^ and Pt^2+^ is 0.36:1 based on Pt 4f_7/2_ and 0.58:1 based on Pt 4f_5/2_ as shown in Supplementary Fig. 13, suggesting that Pt NPs are more negatively charged on h-BNNS than those on SiO_2_ support owing to the interfacial charge transfer between Pt and *h*-BNNS with vacancies. Meanwhile, the Pt^0^ BE in Pt/*h*-BNNS shifted negatively by 0.32 eV compared with that of the Pt/SiO_2_, demonstrating Pt is electron enriched in Pt/*h*-BNNS^42^.

In summary, we have discovered the interfacial electronic effects between Pt and a non-redox active vacancy-abundant *h*-BNNS support. We found that the Pt NPs were embedded on both Nv and Bv and the overall charge transfer is from *h*-BNNS to Pt. Consequently, O_2_ binds stronger than CO molecules, alleviating the CO poisoning of the Pt NPs and promoting the catalysis. This finding may provide a way to design and develop highly stable supported Pt catalysts with controllable activity and selectivity for other heterogeneous catalysis.

## Methods

### Preparation of *h*-BNNS

In a typical preparation process of *h*-BNNS, the commercial *h*-BN (parent bulk *h*-BN, lateral size 1 μm) powder (1 g) in quartz boat was ramped to 800 °C in a muffle furnace under air, kept at this temperature for 5 min, and then immediately immersed into a dewar bottle containing liquid N_2_ (L–N_2_) until the L–N_2_ gasified completely. The above steps were performed repeatedly. The resulting *h*-BNNS was collected by the following process: the as-prepared h-BNNS were dispersed in isopropanol and sonicated for 30 min. Then the dispersion was centrifuged at 800 r.p.m. for 10 min to remove any remaining bulk crystals. The supernatant was collected and dried in vacuum oven overnight.

### Preparation of Pt NPs

Pt(acac)_2_ (0.1 g), 1-octadecene (10 ml), oleic acid 1 ml and oleylamine (1 ml) were mixed under N_2_ and magnetic stirring. The mixture was then heated to 65 °C to dissolve Pt(acac)_2_. The temperature was then raised to ∼160 ^o^C. A solution of Fe(CO)_5_ in hexane (0.1 ml, prepared by adding 0.1 ml Fe(CO)_5_ in 1 ml hexane under N_2_) was injected into the hot solution under N_2_ blanket. The solution was further heated to 200 °C and kept at this temperature for 1 h before it cooled down to room temperature. In total, 40 ml of isopropanol was added and then suspension was centrifuged (8,000 r.p.m.,∼10 min) to separate the Pt NPs. The particles were dispersed in 10 ml hexane and precipitated out by adding ethanol. The process was repeated one more time to purify the NPs. The as-prepared product was dispersed in 10 ml hexane for next use.

### Preparation of Pt/*h*-BNNS catalysts

A certain volume of Pt NPs hexane solution was blow-dried by N_2_, weighing the mass of Pt NPs. A certain amount of *h*-BNNS (mass ratio of Pt NPs and *h-*BNNS is 1:12) was dispersed in the mixture of ethanol (5 ml) and hexane (5 ml) under sonication. Pt NPs were re-dispersed in the hexane (5 ml) under sonication and dropped slowly into the *h*-BNNS solution, sonicating for 1 h. The as-prepared sample was separated via centrifugation, washed with ethanol three times and dried in vacuum at 50 °C for further use. Pt content was 1.18 wt.% by inductively coupled plasma optical emission spectroscopy determination. The details for the preparation of Pt/bulk *h*-BN, Pt/TiO_2_, Pt/SiO_2_ and Pt/C catalysts were presented in supplementary information.

### Catalyst characterisation

AFM was performed on Cypher AFM (Asylum Research) equipped with Nanosensor PPP-EFM probes, which typically have a moderate stiffness of 2–5 N m^−1^; TEM was performed using a Zeiss Libra 120 system operating at 120 kV. Quantitative elemental analysis was done on an inductively coupled plasma optical emission spectrometry on a Perkin-Elmer Optima 2000 DV ICP spectrometer. XPS data were collected using a PHI 3056 spectrometer with an Al anode source operated at 15 kV and an applied power of 350 W with samples mounted on indium foil. The higher energy species in the Pt data corresponds to Indium 4p from the substrate (onset 78 eV), which were unavoidable due to the samples adhesion to the indium foil. Annular dark-field imaging and EELS analysis were carried out using a Nion UltraSTEM100 operated at 60 kV under ultrahigh vacuum (1 × 10^−9^ torr). The microscope is equipped with a cold field-emission gun and an aberrations corrector for sub-angstrom resolution. An inner angle no smaller than 30 mrad was used for annular dark-field imaging. X-ray diffraction was performed on a Panalytical Empyrean diffractometer with Cu K-alpha radiation *λ*=1.5418 Å operating at 45 kV and 40 mA. *In situ* DRIFTS measurement was performed using a Nicolet Nexus 670 spectrometer equipped with a MCT detector cooled by liquid nitrogen. Each spectrum was recorded with 32 scans at a resolution of 4 cm^−1^. The sample was loaded in a porous ceramic cup and inserted into an *in situ* chamber (HC-900, Pike Technologies). The catalyst was pre-treated in the chamber at 200 °C for 1 h in flowing 5% O_2_/He before cooling down to room temperature for CO adsorption.

### General procedure for CO oxidation

CO oxidation was carried out in a temperature-controlled microreactor (Altamira AMI 200) equipped with an on-line gas chromatograph. All experiments were performed under atmospheric pressure with a flow rate of 10 ml min^−1^ of 1% CO balanced in dry air. For the kinetic measurements, the amount of Pt/*h*-BNNS and Pt/SiO_2_ was reduced to 5 mg, respectively, to ensure the CO conversion is below 15%. The CO conversion was averaged at 10, 20, 30 and 40 min to calculate the reaction rate. The reaction rate (*r*) was calculated as equation (1):


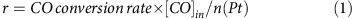


Here, CO conversion rate is the percentage of CO oxidized to CO_2_ after the reaction. [CO]_in_ is the total molar flow of CO per second. n(Pt) stands for total moles of Pt atoms.

### DFT calculations

Spin-polarized periodic DFT calculations were performed using the Vienna Ab initio Simulation Package^43^,^44^ to optimize the Pt_4_ clusters, CO, O_2_ adsorptions and analyse the Bader charge of Pt/h-BNNS models. The *h*-BNNS was modelled using a *p*(5 × 5) supercell, with a vacuum of 20 Å along the direction perpendicular to the substrate. The Generalized Gradient Approximation (GGA)-PBE functional^45^ was used for electronic exchange-correlation effect. The projector wave augmentation method^46^ was used to describe the electron-ion interaction, with a plane wave cutoff energy of 400 eV. A 4 × 4 × 1 k-points mesh based on the Monkhorst-Pack scheme^41^ was used for Brillouin-Zone integration. BEs on clean *h-*BNNS were calculated as: BE=E _Pt4/*h*-BNNS_−E _Pt4_−E _clean *h*-BNNS_; BE on Nv/Bv on *h-*BNNS are calculated as: BE=E_Pt4 on Nv/Bv(*h*-BNNS)_−E_Pt4_−E_Nv/Bv(*h*-BNNS)_. Respective BEs of CO and O_2_ were calculated according to: BE_CO/O2*_=E_CO/O2*_−E_Pt4 on *h*-BNNS_−E_CO/O2(g)._

### Data availability

The authors declare that the data supporting the findings of this study are available from the corresponding author on reasonable request.

## Additional information

**How to cite this article:** Zhu, W. *et al*. Taming interfacial electronic properties of platinum nanoparticles on vacancy-abundant boron nitride nanosheets for enhanced catalysis. *Nat. Commun.* **8,** 15291 doi: 10.1038/ncomms15291 (2017).

**Publisher's note:** Springer Nature remains neutral with regard to jurisdictional claims in published maps and institutional affiliations.

## Supplementary Material

Supplementary Information Supplementary Figures, Supplementary Table, Supplementary Methods and Supplementary References

Supplementary Movie Electron energy loss spectroscopy (EELS) to map the individual B and N in the Pt/h-BNNS sample, observing a chemical shift of B-K edge in its pre-peak depending only on the distance to the Pt particles.

## Figures and Tables

**Figure 1:**
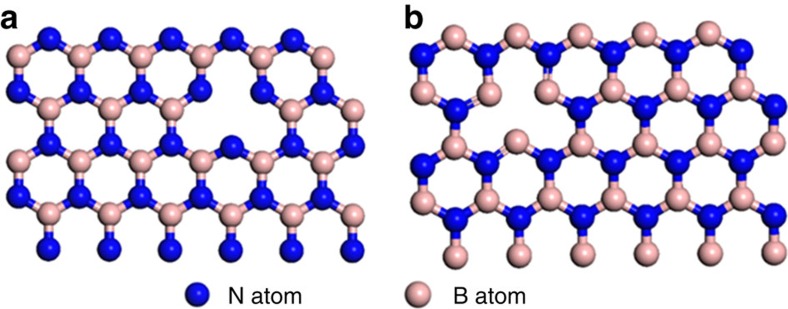
Schematic illustration of *h*-BNNS with B and N vacancies. (**a**) *h*-BNNS with B vacancy and N-terminated edge. (**b**) *h*-BNNS with N vacancy and B-terminated edge.
