# Supplementary material for: Taming interfacial electronic properties of platinum nanoparticles on vacancy-abundant boron nitride nanosheets for enhanced catalysis
Source: Nat Commun. 2017 Jun 9;8:15291. doi: 10.1038/ncomms15291 (PMC5472761; doi:10.1038/ncomms15291)
Supplement: Supplementary Information — Supplementary Figures, Supplementary Table, Supplementary Methods and Supplementary References [file ncomms15291-s1.pdf]

Supplementary Table 1| Comparison between the performance of Pt/h-BNNs and literature reported catalysts.

Catalysts	Pt loadings	SV(Lg^{-1}Pt h^{-1})	T_0	T_{100}	Note
Pt/SiO_2	1 wt.%	874	150°C	200°C	[1]
Pt/CNT	1 wt.%	990	-	180°C	[2]
Pt/Al_2O_3	50 g ft^{-3}	3205	80°C	150°C	[3]
Pt/Al_2O_3	1.03 wt.%	-	100°C	180°C	[4]
Pt/h-BNNS	1.18 wt.%	1667	35°C	67°C	This work

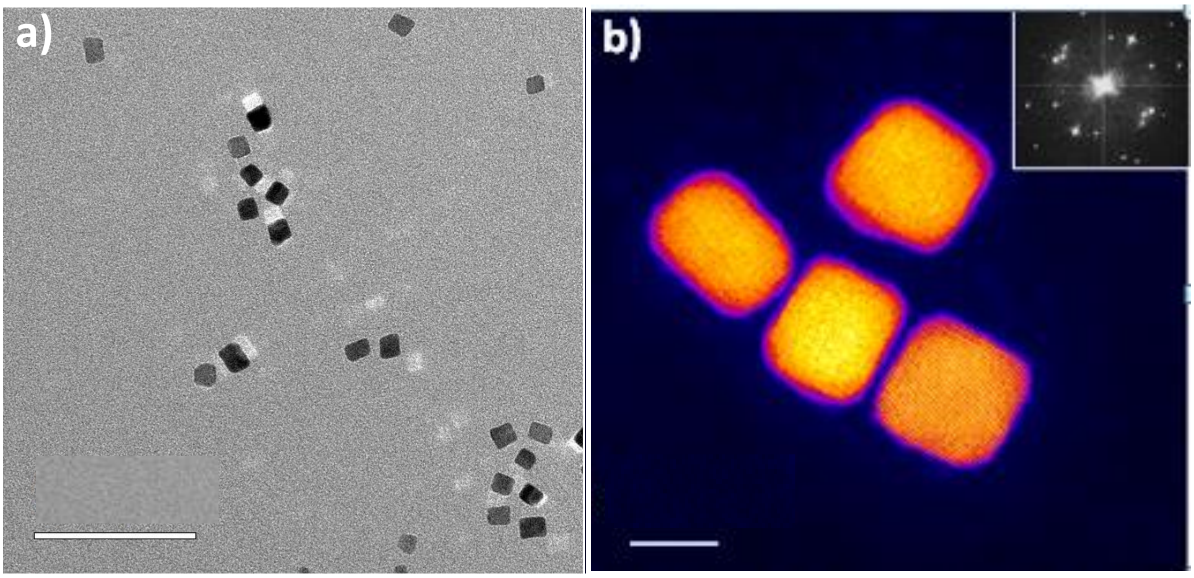

Supplementary Figure 1 | TEM (**a**: Scale bar, 50 nm) and STEM (**b**: Scale bar, 5 nm.) of Pt NPs (inset is the electron diffraction pattern of Pt NPs).

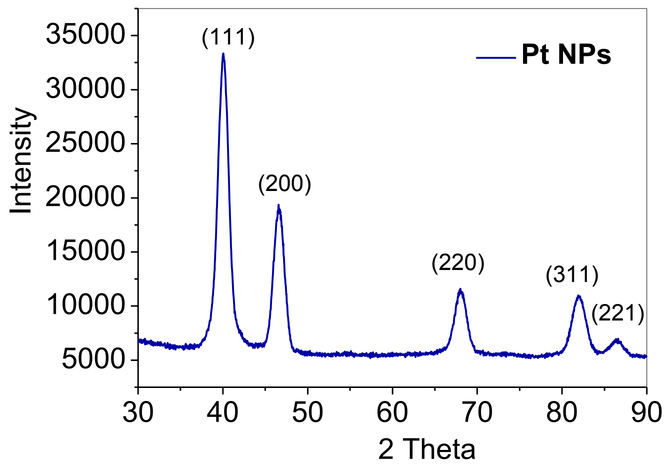

Supplementary Figure 2| XRD of Pt NPs.

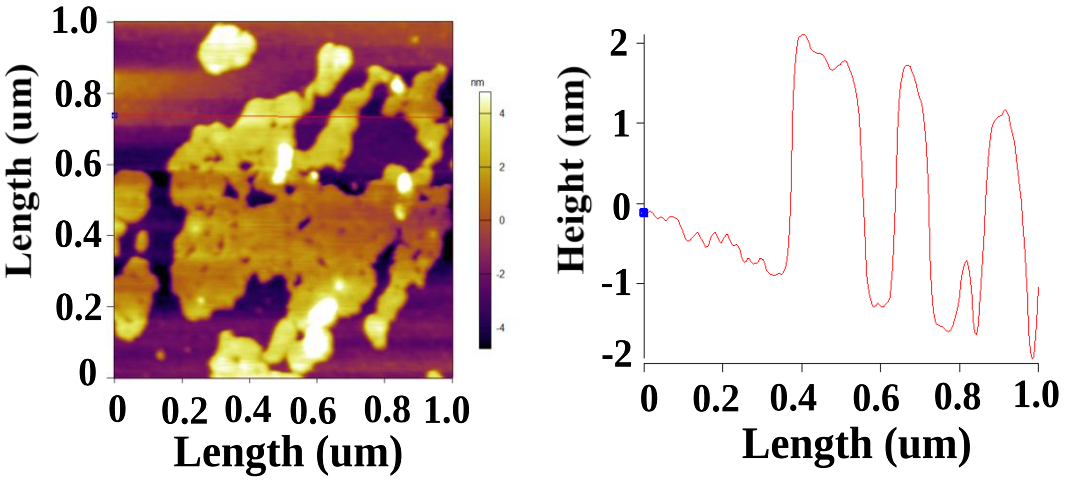

Supplementary Figure 3 | AFM image and the corresponding height profiles of *h*-BNNS

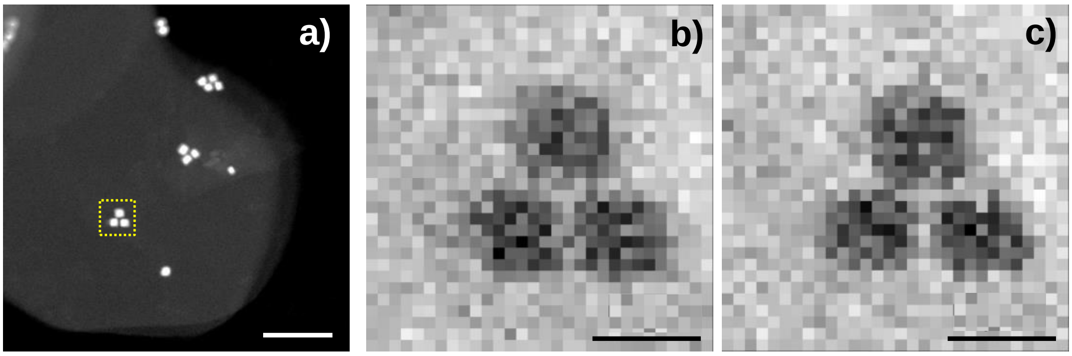

Supplementary Figure 4 | a) ADF image showing the distribution of bright Pt NPs in Pt/*h*-BNNS. Scale bar, 50 nm. **b**) B-K, Scale bar, 10 nm. c) N-K EELS elemental mapping obtained from the local area marked by a dotted square in (**a**), Scale bar, 10 nm.

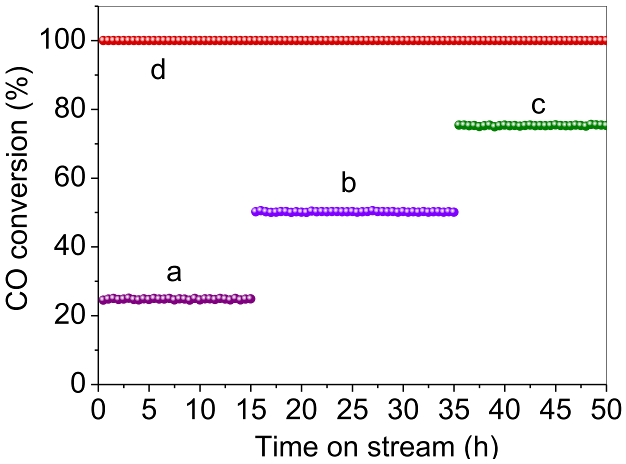

Supplementary Figure 5 | CO conversion of Pt/h-BNNS at different temperatures a: 39°C b:45 ^{o}C c:49 ^{o}C d:75 ^{o}C, m（catalyst）=30 mg   Pt content= 1.18 wt.%

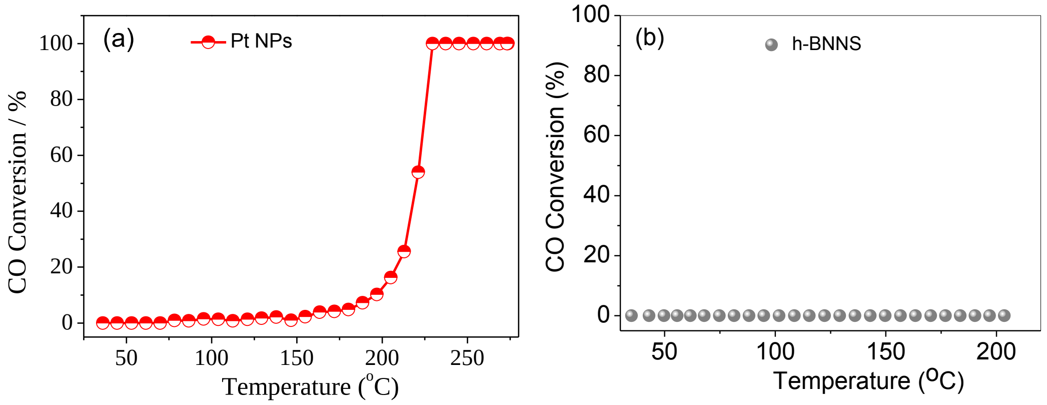

Supplementary Figure 6 | CO oxidation activity of Pt NPs (**a**) and *h*-BNNS (**b**) alone. The mass of the catalyst equals with the amount of Pt NPs and h-BNNS in 30 mg Pt NPs/*h*-BNNS, respectively. CO flow rate 10 mL min^{-1};

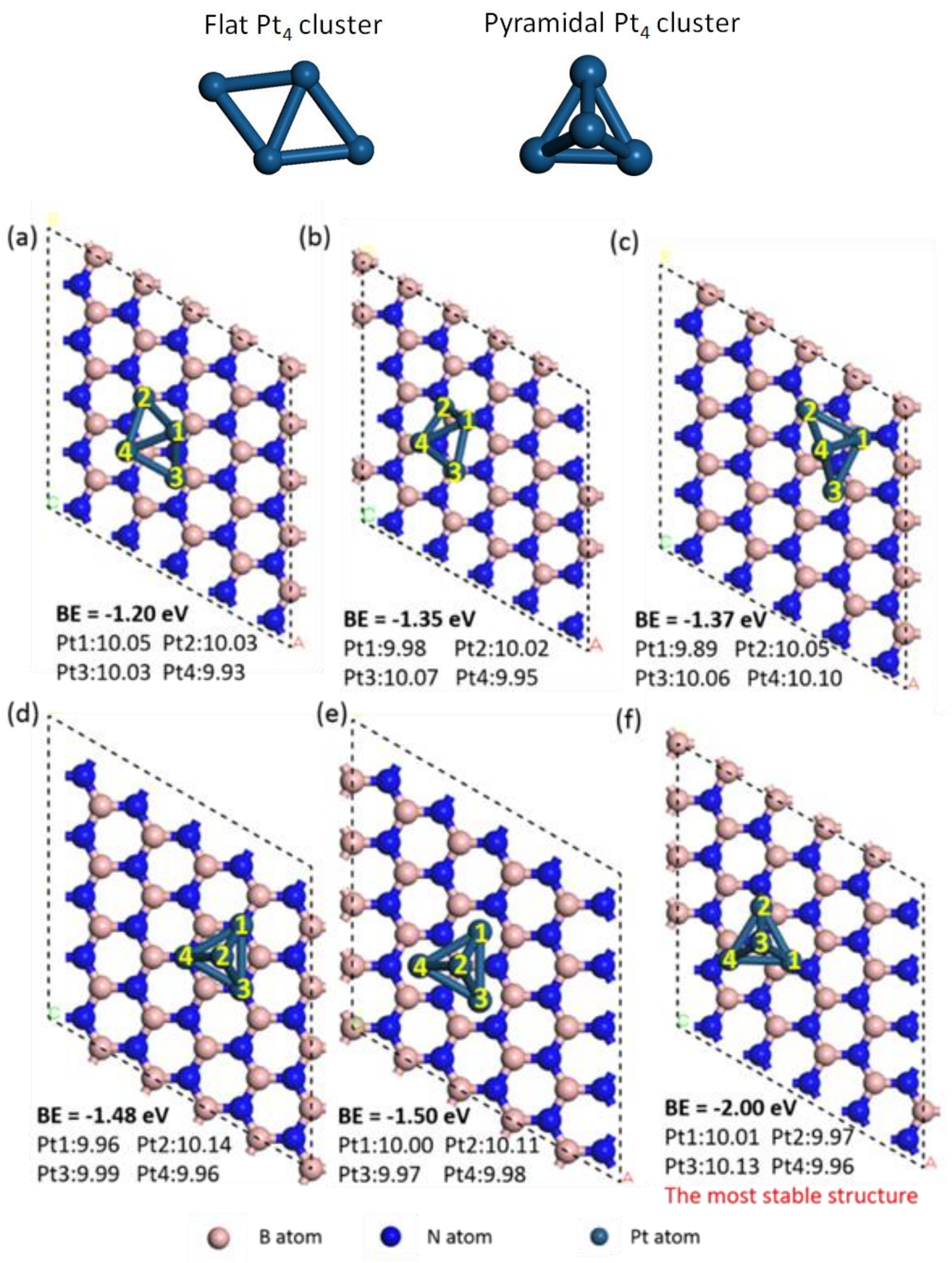

Supplementary Figure 7 | Optimized structure and corresponding Bader charge of a Pt_4 cluster on clean *h*-BNNS considered in this study. (**a-c**) Flat Pt_4 cluster on *h*-BNNS. (**d-f**) Pyramidal Pt_4 cluster on *h*-BNNS.

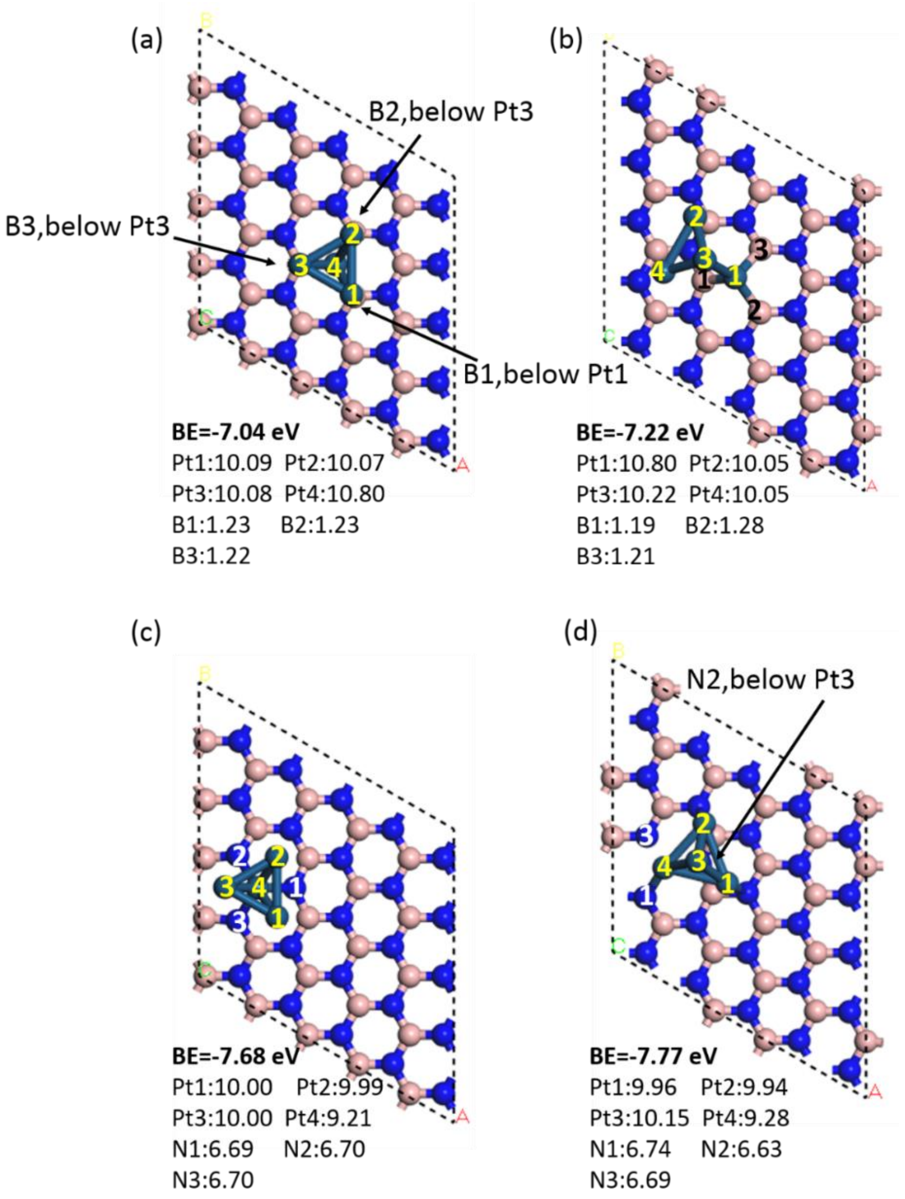

Supplementary Figure 8 | Optimized structure and corresponding Bader charge of a Pt_4 cluster on (**a**) Nv (**b**) Nv (**c**) Bv (**d**) Bv *h*-BNNS considered in this study

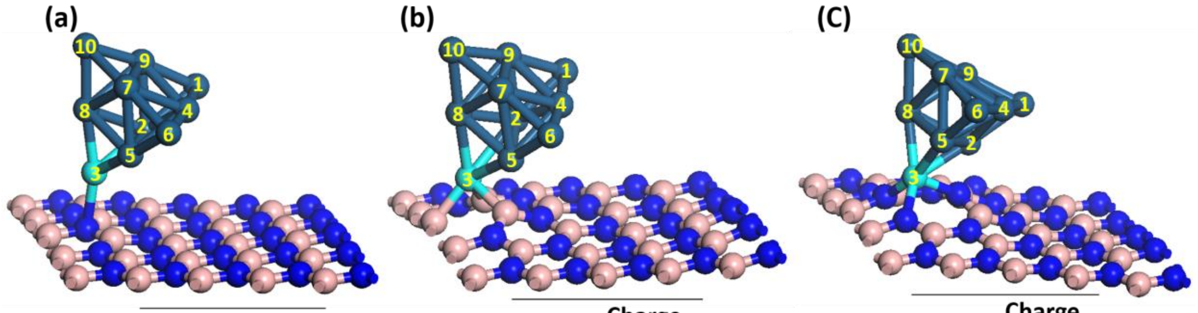

(a)

	Charge
Pt1	+0.17
Pt2	-0.08
Pt3	+0.11
Pt4	-0.10
Pt5	-0.10
Pt6	+0.17
Pt7	-0.09
Pt8	-0.07
Pt9	-0.09
Pt10	+0.17
sum	+0.08

(b)

	Charge
Pt1	+0.16
Pt2	-0.03
Pt3	+0.76
Pt4	-0.09
Pt5	-0.01
Pt6	+0.16
Pt7	-0.06
Pt8	+0.02
Pt9	-0.08
Pt10	+0.17
sum	+0.98

(C)

	Charge
Pt1	+0.16
Pt2	-0.18
Pt3	-0.62
Pt4	-0.09
Pt5	-0.05
Pt6	+0.15
Pt7	-0.08
Pt8	-0.02
Pt9	-0.07
Pt10	+0.18
sum	-0.62

Supplementary Figure 9 | Optimized structures and Bader charges of Pt_{10} cluster on *h*-BNNS. (**a**) Pt_{10} cluster on clean, vacancy-free *h*-BNNS, (**b**) Pt_{10} cluster *h*-BNNS with Nv, and (**c**) Pt_{10} cluster on *h*-BNNS with Bv. The sum here is the total valence electrons for Pt_{10} cluster. The Pt atoms with the most charge transfer on *h*-BNNS with Nv and Bv are highlighted in blue. '+' sign represents charge accumulation; while '-' sign represents charge depletion.

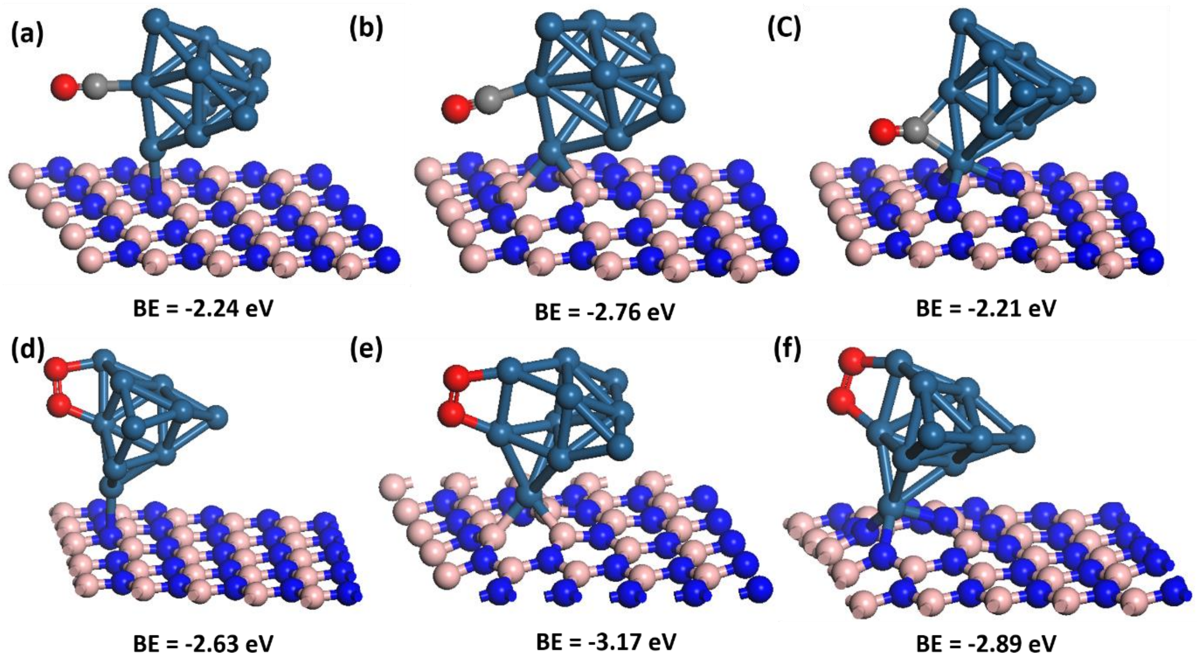

Supplementary Figure 10 | The most stable configuration of CO (**a-c**) and O_2 (**d-f**) adsorption and BEs on Pt_{10} cluster. (**a**) Pt_{10} cluster on clean, vacancy-free *h*-BNNS, (**b**) Pt_{10} cluster *h*-BNNS with Nv, and (**c**) Pt_{10} cluster on h-BNNS with Bv, (**d**) Pt_{10} cluster on clean, vacancy-free *h*-BNNS, (**e**) Pt_{10} cluster *h*-BNNS with Nv, and (**f**) Pt_{10} cluster on *h*-BNNS with Bv.

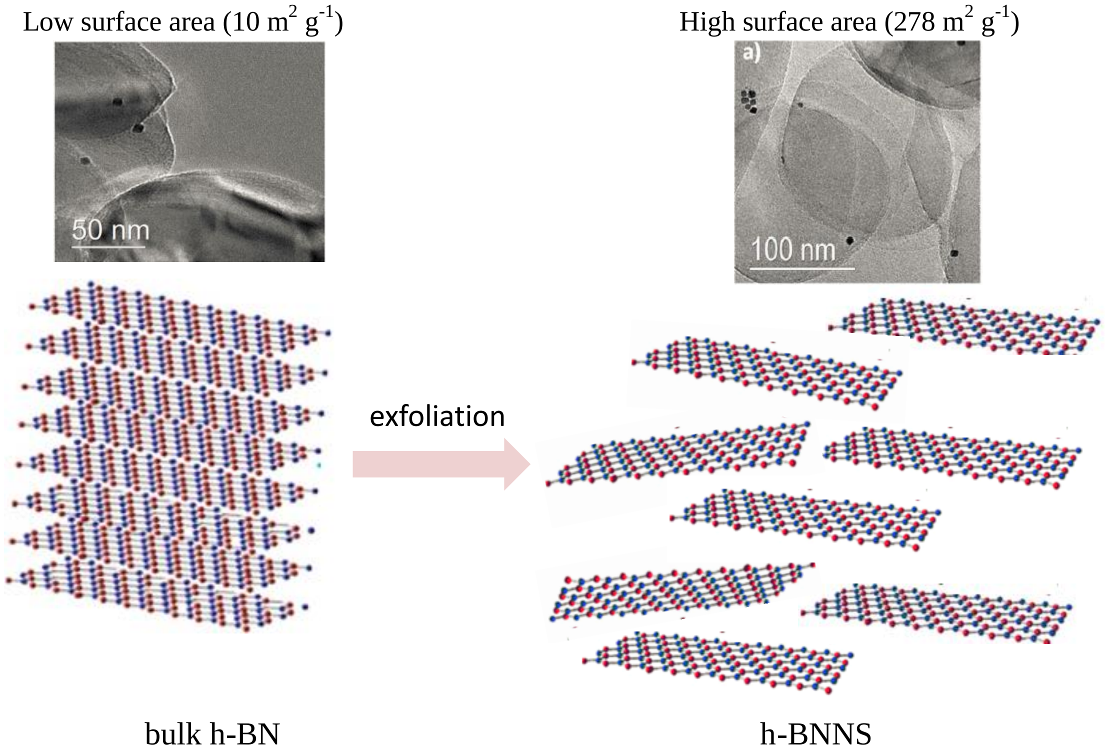

Supplementary Figure 11 | Pt NPs supported on bulk BN and *h*-BNNS and the schematic illustration of the exfoliation of bulk BN to *h*-BNNS.

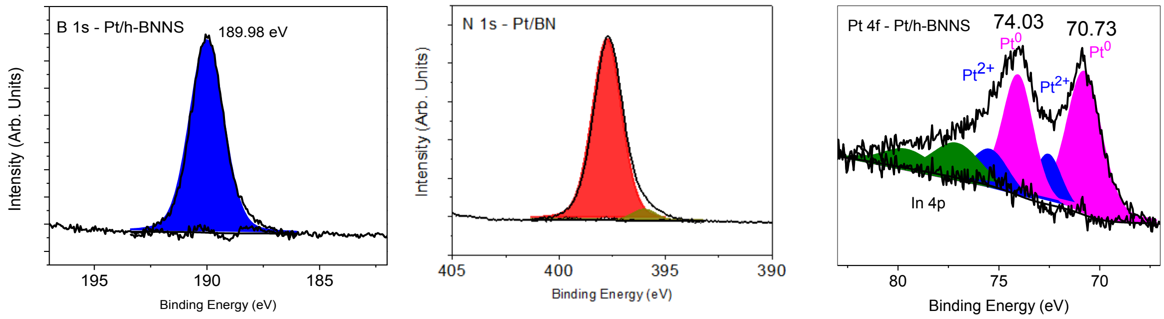

Supplementary Figure 12 | XPS of Pt/h-BNNS

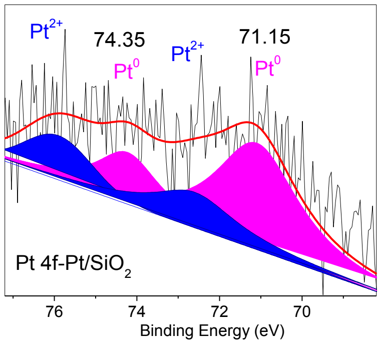

Supplementary Figure 13 | XPS of Pt/SiO_2

Supplementary Methods

Preparation of Pt/bulk BN catalysts. A certain volume of Pt NPs hexane solution was blow-dried by N_2, weighing the mass of Pt NPs. A certain amount of bulk BN (mass ratio of Pt NPs and bulk BN is 1:11) was dispersed in the mixture of ethanol (5 mL) and hexane (5 mL) under sonication. Pt NPs were re-dispersed in the hexane (5 mL) under sonication and dropped slowly into the bulk BN solution, sonicating for 1 h. The as-prepared sample was separated via centrifugation, washed with ethanol three times and dried in vacuum at 50℃ for further use. Pt content in Pt/bulk BN was 1.21 wt.% by ICP-OES.

Preparation of Pt/TiO_2 catalysts. A certain volume of Pt NPs hexane solution was blow-dried by N_2, weighing the mass of Pt NPs. A certain amount of P25 (mass ratio of Pt NPs and P25 is 1:11) was dispersed in the mixture of ethanol (5 mL) and hexane (5 mL) under sonication. Pt NPs were re-dispersed in the hexane (5 mL) under sonication and dropped slowly into the P25 solution, sonicating for 1 h. The as-prepared sample was separated via centrifugation, washed with ethanol three times and dried in vacuum at 50℃ for further use. Pt content in Pt/TiO_2 was 1.15 wt.% by ICP-OES.

Preparation of Pt/SiO_2 catalysts. A certain volume of Pt NPs hexane solution was blow-dried by N_2, weighing the mass of Pt NPs. A certain amount of SiO_2 (mass ratio of Pt NPs and SiO_2 is 1:12) was dispersed in the mixture of ethanol (5 mL) and hexane (5 mL) under sonication. Pt NPs were re-dispersed in the hexane (5 mL) under sonication and dropped slowly into the SiO_2 solution, sonicating for 1 h. The as-prepared sample was separated via centrifugation, washed with ethanol three times and dried in vacuum at 50℃ for further use. Pt content in Pt/SiO_2 was 1.16 wt.% by ICP-OES.

Preparation of Pt/C catalysts. A certain volume of Pt NPs hexane solution was blow-dried by N_2, weighing the mass of Pt NPs. A certain amount of acetylene black (mass ratio of Pt NPs and acetylene black is 1:12) was dispersed in the mixture of ethanol (5 mL) and hexane (5 mL) under sonication. Pt NPs were re-dispersed in the hexane (5 mL) under sonication and dropped slowly into the acetylene black solution, sonicating for 1 h. The as-prepared sample was separated via centrifugation, washed with ethanol three times and dried in vacuum at 50℃ for further use. Pt content in Pt/C was 1.18 wt.% by ICP-OES.

Supplementary REFERENCES

[1] Jung, C. *et al.* Catalytic activity of Pt/SiO_2 nanocatalysts synthesized via ultrasonic spray pyrolysis process under CO oxidation, *Appl. Catal. B-Environ.*, **154–155**, 171–176 (2014).

[2] Jardim, E. O., Gonçalves, M., Rico-Francés, S., Sepúlveda-Escribano, A., & Silvestre-Albero, J. Superior performance of multi-wall carbon nanotubes as support of Pt-based catalysts for the preferential CO oxidation: Effect of ceria addition, *Appl. Catal. B- Environ.*, **113–114**, 72–78 (2012).

[3] Hazlett, M. J., & Epling, W. S. Spatially resolving CO and C_3H_6 oxidation reactions in a Pt/Al_2O_3 model oxidation catalyst, *Catal. Today*, **267**, 157–166 (2016).

[4] Ivanova, A.S. *et al*. Metal–support interactions in Pt/Al_2O_3 and Pd/Al_2O_3 catalysts for CO oxidation, *Appl. Catal. B-Environ.*, **97**, 57–71 (2010).
